# Additively manufactured titanium scaffolds and osteointegration - meta-analyses and moderator-analyses of in vivo biomechanical testing

**DOI:** 10.1186/s40824-021-00216-8

**Published:** 2021-06-10

**Authors:** Simon Cleemput, Stijn E. F. Huys, Robbert Cleymaet, Wilfried Cools, Maurice Y. Mommaerts

**Affiliations:** 1 Doctoral School of Life Sciences and Medicine, Vrije Universiteit Brussel, 1090 Brussels, Belgium; 2 European Face Centre, Universitair Ziekenhuis Brussel, Vrije Universiteit Brussel, 1090 Brussels, Belgium; 3 Engineering Science, Department of Mechanical Engineering, Section of Biomechanics, Catholic University of Leuven, 3000 Leuven, Belgium; 4 Interfaculty Center Data processing and Statistics, Vrije Universiteit Brussel, 1090 Brussels, Belgium

**Keywords:** 3D printing, Titanium, Animal experimentation, Meta-analysis

## Abstract

**Introduction:**

Maximizing osteointegration potential of three-dimensionally-printed porous titanium (3DPPT) is an ongoing focus in biomaterial research. Many strategies are proposed and tested but there is no weighted comparison of results.

**Methods:**

We systematically searched Pubmed and Embase to obtain two pools of 3DPPT studies that performed mechanical implant-removal testing in animal models and whose characteristics were sufficiently similar to compare the outcomes in meta-analyses (MAs). We expanded these MAs to multivariable meta-regressions (moderator analysis) to verify whether statistical models including reported scaffold features (e.g., “pore-size”, “porosity”, “type of unit cell”) or post-printing treatments (e.g., surface treatments, adding agents) could explain the observed differences in treatment effects (expressed as shear strength of bone-titanium interface).

**Results:**

“Animal type” (species of animal in which the 3DPPT was implanted) and “type of post-treatment” (treatment performed after 3D printing) were moderators providing statistically significant models for differences in mechanical removal strength. An interaction model with covariables “pore-size” and “porosity” in a rabbit subgroup analysis (the most reported animal model) was also significant. Impact of other moderators (including “time” and “location of implant”) was not statistically significant.

**Discussion/conclusion:**

Our findings suggest a stronger effect from porosity in a rat than in a sheep model. Additionally, adding a calcium-containing layer does not improve removal strength but the other post-treatments do. Our results provide overview and new insights, but little narrowing of existing value ranges. Consequent reporting of 3DPPT characteristics, standardized comparison, and expression of porosity in terms of surface roughness could help tackle these existing dilemmas.

**Graphical abstract:**

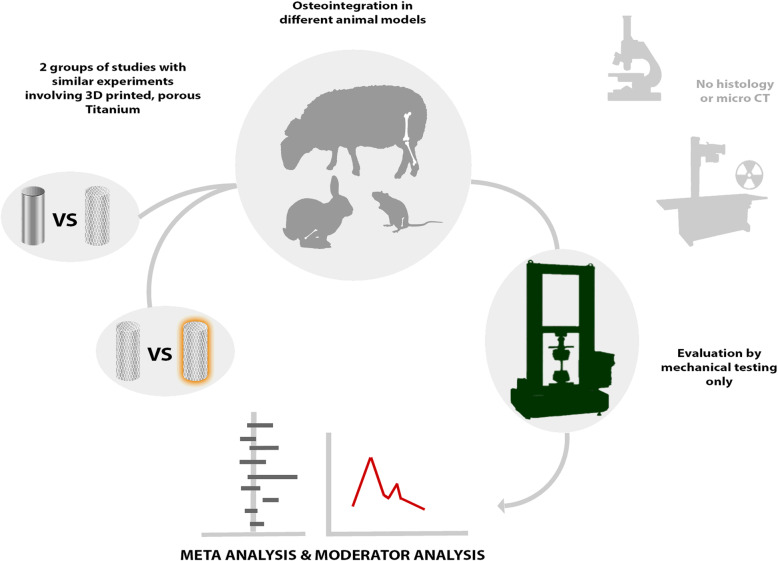

## Background

Regarding biocompatibility and appropriate biomechanical behavior, titanium (Ti) and its alloys surpass most other metals used for medical implantation purposes. The stabile passivation of Ti into TiO_2_ makes implants comprising Ti and Ti alloys well shielded from the surrounding tissue, preventing them from further corrosion and protecting the surrounding tissue from possible toxicity [1]. Additionally the high fatigue and tensile strength at a low density and low elastic modulus make constructs comprising Ti6Al4V, TiALNb, and the higher grade commercially pure Ti (CP gr 3 and 4) less prone to causing stress-shielding and suitable for osteointegration (OI) subject to dynamic loading [2]. The commercial viability of 3D metal printing has emerged in the last decade. Because this production method is often applied to fabricate light, thin, intricate structures, the search for an appropriate printing metal has also granted Ti a favorable position. For implantation purposes, 3D printing using titanium means that implants can now be fabricated in a highly personalized manner because of a digital workflow that starts from high-resolution images that are often readily available (e.g., medical CT scans). Another advantage (a consequence of the “additive” instead of more conventional “subtractive” approach) of 3D printing is that constructs can now be fabricated that are fully and internally porous, with open, interconnected pores and reaching the full internal depth of the construct.

Although the primary intent to print titanium implants porously was to further lower the stiffness and thus the possible stress-shielding effect (Ti6Al4V has a Young’s modulus of 104 GPa, still approximatively 5 times higher than that of cortical bone (20 GPa) and 10 times higher than that of trabecular bone (10 GPa) [3]), the porous surface is also believed to aid in OI. Much like the porous metal outer layers made with conventional, subtractive production methods (such as powder metallurgy using space-holding agents), the 3D-printed pores provide space for the surrounding bone tissue to grow into and add contact surface (or surface roughness) to the construct. This principle is established and has been applied in medical devices such as cementless hip prostheses, spinal fusion cages, and dental implants for almost 2 decades. Achieving total interconnectivity of the individual pores and control of the exact number (= “porosity”), dimensions (= “pore-size”), and shape (= “unit cell”) of the pores is a new given.

3DPPT has the potential to evolve into one of the most successful OI strategies, and the research field is thriving. The literature reporting on well toughed-through lattice designs and enhancing post-printing treatments is rapidly increasing, aiming to achieve a maximal OI. However, the designs and treatments are becoming increasingly specific and are often tested in vitro or in very specific in vivo models, making it difficult to draw conclusions that apply to 3DPPT in general, or to slightly different designs or models. Thus, regarding the importance of 3DPPT features, namely lattice design parameters (e.g., “pore-size”, “porosity”, and “type of unit-cell”) and effectiveness of applied post-printing treatments (e.g., coatings and surface treatments), the current literature offers broad ranges of values and few consensuses (examples listed in the Discussion section). However, none of these data are sharply defined and none are the result of any systematic gathering and weighted comparison of outcomes.

Considering that the literature is becoming extensive and that several uncertainties remain, we subjected 3DPPT to statistical analysis. Unmistakably realizing the difficulty in making valid comparisons in this research field, we conducted a systematic literature review (SR) with a meta-analysis (MA) and meta-regression (moderator-analysis) to respond to our research questions “How do the results of studies evaluating the OI of different 3DPPT designs and treatments compare to each other, and to which extent are differences in the results statistically linked to design parameters or post-printing treatments?”

Considering all possible approaches and to provide an overview of tangible OI results, we evaluated only in vivo animal studies, focusing on OI evaluation using mechanical removal testing.

## Main text

### Methods

Our systematic review and meta-analyses were performed according to the CAMARADES (“Collaborative Approach to Meta-Analysis and Review of Animal Data from Experimental Studies) guidelines (http://www.dcn.ed.ac.uk/camarades/default.htm) [4]. Our protocol was registered at PROSPERO (https://www.crd.york.ac.uk/prospero/display_record.php?RecordID=211733), and we reported the information according to the PRISMA 2009 checklist.

#### Search strategy

We searched the electronic databases of Pubmed (Medline) and Embase with the search terms listed in Table 1 connected by the Boolean search words. Being careful not to overlook any animal models, we did not include an exhaustive list of animals, but rather manually selected the in vivo studies from our search results.

**Table 1:**
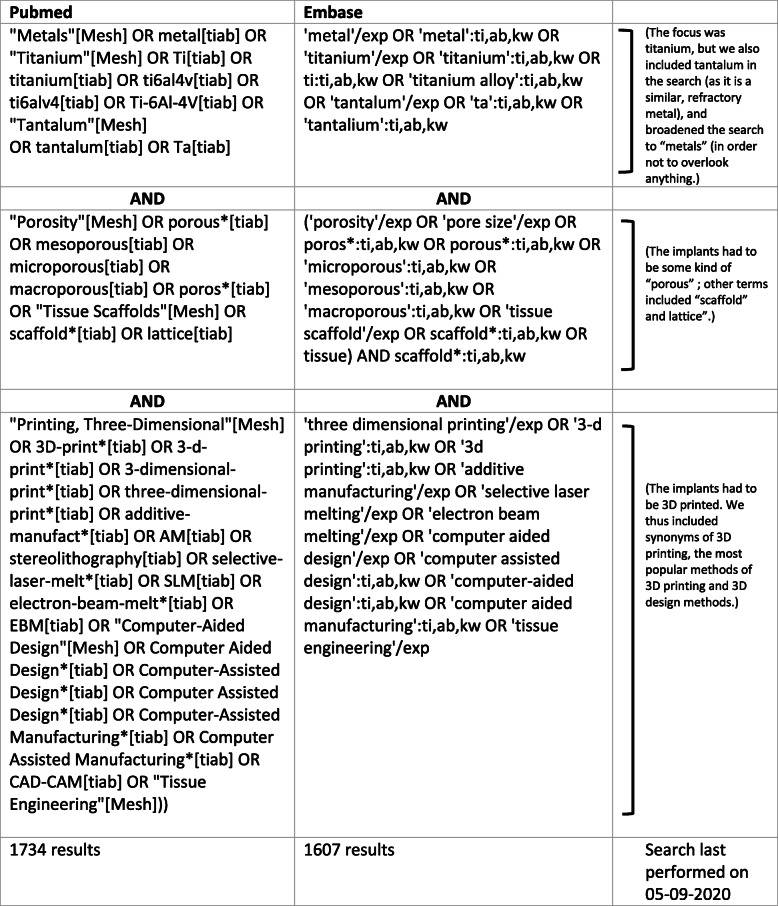
Literature search

#### Study selection

We exported the literature search into Endnote X9 and removed duplicates. After that, study selection was performed using Rayyan online software, applying a 3-step approach. We included original animal studies (analytical, experimental research) that evaluated OI of 3DPPT (or Tantalum) implants mechanically in a measurable, quantitative way. We exclude studies that only performed histomorphological or radiological evaluation. Next, we selected the studies that performed implant removal testing (push-out, pull-out, or torque-out). We excluded studies using three-point bending and range of motion (ROM) testing because they do not allow for clear identification of the forces applied to the bone-implant interface. The remaining studies were divided into two pools to perform two separate MAs. The studies were allocated based on the type of intervention performed but must be comparable to a very similar control group. MA1 comprises studies that compared the OI of an experimental group (Exp) of 3DPPT against a control group (Co) of solid, non-porous, 3D printed titanium.^1^ MA2 comprises studies that compared the OI of an experimental group (Exp) of post-treated 3DPPT against a control group (Co) of non-post treated 3DPPT.^2^

#### Data extraction

We read the full-text articles of the obtained publications and gathered the following information: study characteristics (author, title, and year of publication); implant shape and dimensions; characteristics of the 3D-printed porous structure (pore-size, porosity, and unit cell); 3D printing method; type of titanium (alloy) used; post-printing treatment; animal model used; site of implantation, type of bone-tissue present at the site; method of implantation; and fit of the implant in the bone.

We also gathered the reported outcomes of the OI evaluation, which, depending on the type of mechanical testing performed, were the peak removal force or load (N) (when the implant was pushed or pulled out of a resected bone specimen), peak removal torque (Nmm) or torsion force (N/cm) (in the case of torque-out testing) and shear strength or modulus (Mpa) (the peak removal force or load divided by the bone-titanium interface area subject to the removal force).

The selection and data extraction processes were performed by two researchers (RC and SC) who worked independently and resolved discrepancies by consensus. Whenever desired information was not reported, the authors of the publication were contacted and requested to submit information by e-mail. Twenty-four authors were e-mailed from March 21 to May 18, 2020. Whenever a request for an outcome value was not met, online measuring software (https://apps.automeris.io/wpd/) was used to derive an estimated value from published graphs. This measuring process was also independently performed by these two researchers (RC and SC), and the final values used for statistical analyses were the calculated means of their estimations.

#### Data synthesis and statistical method

We performed two separate MAs of aggregate data of the included mechanical testing using implant removal studies. We chose to convert all the obtained outcomes and standard deviations (SDs) that were not already expressed as such to the common unit “shear strength” (Mpa) because this unit balances discrepancies caused by differences in size—that is, the contact surface of the implants. For this task, we applied Eq. 1), with force (F) representing the obtained outcome or SD and area (A) representing the shear surface of the implant and bone during implant removal testing. The shear surface (A) itself was calculated from the obtained “implant dimensions” (height and diameter) according to Eq. 2), most often as the mantle surface of the implant (perpendicular to the direction of the removal force applied) and always in strict accordance with the study specifications (considering the depth of implantation or possible non-geometric shape). In the case of torque-out testing, (F) was calculated according to Eq. 3), as the torque value (tau) divided by the radius of the implant (cylinder or screw).

Formulae and equations

1 $$ shear\kern0.17em strenght= force/ surface\kern0.5em SS=F/A $$

2 $$ {A}_{cylinder, screw, prism\kern0.34em or\kern0.34em block}= base\times height\kern0.5em A=b\times h $$

3 $$ Torque\kern0.17em out\;{force}_{cylinder, screw}= torque/ distance\kern0.5em F= tau/{radius}_{cylinder, screw} $$

From the obtained or converted outcomes and SDs, standardized mean differences (SMDs) and 95% confidence intervals (CIs) were calculated using Hedges’ g method. We used τ^2^ (calculated using the Hartung-Knapp-Sidik-Jonkman (HKSJ) method) and I^2^ to quantify heterogeneity. The obtained MAs were tested for outliers and finally expanded to multivariable meta-regressions (moderator analyses) to examine the effects of the variables “time”, “animal model”, “location of implantation”, “pore-size”, “porosity”, “Struth size” and “type of unit cell” (for MA 1) and “time”, “animal model”, “location of implantation”, and “type of surface treatment” (for MA 2) on the calculated treatment effect (TE).

#### Quality and risk of bias assessment

We used Syrcle’s Risk of Bias tool (ROB) to evaluate the methodological quality of the studies included in the 2 MAs. This tool is derived from Cochrane’s ROB tool and is preferred by CAMARADES [4].

### Results

#### Study selection

**Fig. 1:**
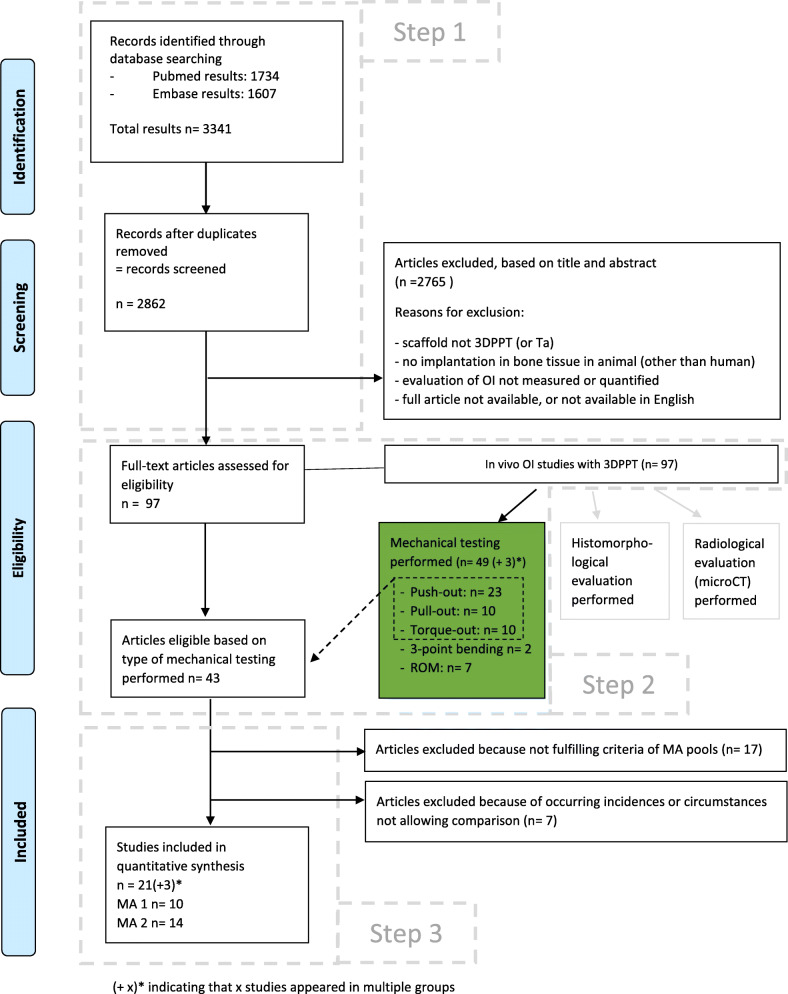
PRISMA flow-chart. (+ x)* indicating that x studies appeared in multiple groups

As shown in Fig. 1, step 1 in our selection process obtained 97 studies, of which 43 remained after step 2. Data extraction allowed the allocation of 26 articles among two MA pools, and further narrowing (to obtain truly comparable groups of studies) brought the total to 10 studies in MA1 [5–14] (providing 32 observations) and 14 studies in MA2 [7–9, 15–24] (providing 40 observations). Three studies were included in both MAs [7–9].

#### Study characteristics

The study characteristics included in MA1 and MA2 are listed in Tables 2 and 3. We included different animal models [rat, rabbit (regular and osteoporotic OVX), dog, and sheep], with sample sizes between 2 and 12 and follow-up periods between 2 weeks and 6 months. Almost all the studies used a trabecular bone model (femur condyle, pelvis, and tibia), except for [8], which used a cortical (calvaria onlay) model (with perforations of the bone at the implantation site). The implants were most often regular and cylindrical (otherwise screw, prism, or block shaped) with reported dimensions, allowing the calculation of the shear surface (A). The implant reported by Amin Yavari et al. was irregularly shaped but was included because the bone-implant contact surface of this construct was a clearly defined circle, which corresponded to the shear surface during the performed torque-out testing [15]. The study reported by Xu et al. was approached similarly because the implant was cup-shaped but with a clearly defined circle as the bone-contact surface, corresponding to the shear surface during pull-out testing [21].

**Table 2:** Characteristics of studies in MA1

Author Publication year	Animal	Implantation site (type of bone)	Implant shape and dimensions (diameter and height)	Controle group (Co) = dense titanium	Experimental group (Exp) = 3DPPT	Experimental group characteristics				Reported outcome of mechnical testing	follow-up (weeks)	Reference Name in MA 1
						Pore size (μm)	Porocity (%)	Struth size (μm)	Unit cell			
Stübinger et al. 2013 [5]	sheep	pelvis (= trabecular)	screw d = 4,1 mm, h = 9 mm	“sandblasted and etched (SE)”	“Additive manufactured (AM)” (= por Ti, sandblasted)	500	50		gyroid; 3 armed with angles of 120°	“Removal torque out (Nmm)” mean + SD reported ( [5]; Table IV)	2	Stübinger (at 2 weeks)
											8	Stübinger (at 8 weeks)
Hara et al. 2016 [6]	rabbit	femur condyle (= trabecular)	cylinder d = 5 mm, h = 12 mm	“Ti-spray” (= melted Ti sprayed onto solid Ti core)	“Porous Ti-alloy (640 μm)”	656 (SD 29)	70 (SD 0,7)		diamond	“Peak load of push-out testing” (N) mean + SD reported ( [6]; page 4, section 3)	4	Hara (at 4 weeks)
											12	Hara (at 12 weeks)
Bandyopadhyay et al. 2017 [7]	rat	femur condyle (= trabecular)	cylinder d = 3 mm, h = 5 mm	“Dense Ti”	“LENS™ Porous Ti” (= “Laser Engineered Net Shaping”)	200–300	25			“Shear modulus (Mpa)”, calculated from push-out testing mean + SD reported ( [7]; Table 3)	4	Bandyopadhyay (at 4 weeks)
											10	Bandyopadhyay (at 10 weeks)
Cheng et al. 2017 [8]	rat	calvaria onlay (= cortical)	block l = 3,5 mm, w = 5 mm h = 2 mm	“Solid” (CaF grit blasted + acid etched)	“Porous” (= por Ti (CaF grit blasted+ acid etched))	563 (SD 2)	67 (SD 3)			“Pull-out force (N) mean + SD measured ( [8]; Fig. 4)	10	Cheng (at 10 weeks)
MacBarb et al. 2017 [9]	sheep	femur condyle (= trabecular)	triangular prism d = 7 mm (inscribed core circle), h = 45 mm	“TPS” (= CP Ti plasma-sprayed onto wrought Ti6AL4V core)	“AM” (= additive manufactured porous Ti)	300	60			“Ultimate Shear Strength (Mpa)” calculated from push-out testing mean + SD measured ( [9]; Fig. 4a)	6	MacBarb (at 6 weeks)
											12	MacBarb (at 12 weeks)
Ran et al. 2018 [10]	rabbit	femur condyl (= trabecular)	cylinder d = 4 mm, h = 13 mm	“dense”	“p500”	401 (SD 26)		300		“Mean interfacial strength (Mpa)” calculated from push-out testing mean + SD measured ( [10]; Fig. 8e)	4	Ran 1 (at 4 weeks)
											12	Ran 1 (at 12 weeks)
					“p700”	607 (SD 24)					4	Ran 2 (at 4 weeks)
											12	Ran 2 (at 12 weeks)
					“p900”	801 (SD 33)					4	Ran 3 (at 4 weeks)
											12	Ran 3 (at 12 weeks)
Wang et al. 2018 [11]	rabbit	femur condyle (= trabecular)	cylinder d = 4,8 mm, h = 8 mm	“Ts” (= “Ti solid”)	“Ti-r” (= “Ti regular distribution”)	457,5 (SD 31,1)	62,5 (SD 2,1)	424 (SD 25,6)	(diamond, regular distribution)	“Push out force (N)” mean + SD measured ( [11]; Fig. 7c)	2	Wang 1 (at 2 weeks)
											4	Wang 1 (at 4 weeks)
											8	Wang 1 (at 8 weeks)
					“Ti-ir” (= “Ti iregular distribution”)	452,5 (SD 43,5)	61,1 (SD 2,2)	435 (SD 36,5)	(diamond, irregular distribution)		2	Wang 2 (at 2 weeks)
											4	Wang 2 (at 4 weeks)
											8	Wang 2 (at 8 weeks)
					“Ti-g” (= “Ti with gradiënt”)	427,0 (SD 40,4)	61,6 (SD 2,8)	449 (SD 34,5)	(diamond, gradiënt distribution)		2	Wang 3 (at 2 weeks)
											4	Wang 3 (at 4 weeks)
											8	Wang 3 (at 8 weeks)
					“Ti-tr” (= “Ti tetrahedral”)	458,5 (SD 29,6)	66,2 (SD 2,0)	410 (SD 23,2)	(tetrahedral)		2	Wang 4 (at 2 weeks)
											4	Wang 4 (at 4 weeks)
											8	Wang 4 (at 8 weeks)
Chang et al. 2019 [12]	rabbit	femur condyle (= trabecular)	screw d = 4,3 mm, h = 10 mm	“DMLS type 4 non-porous”	“DMLS type 6 porous”	300–500	55			“Pull out Strength (N)” mean + SD reported ( [12]; page9, section 3.3)	6	Chang (at 6 weeks)
											12	Chang (at 12 weeks)
Huang et al. 2020 [13]	rabbit	tibia (= trabecular)	screw d = 2,1 mm, h = 9,7 mm	“Control screw”	“AMD interference screw” (= por Ti + 1 μm thick HA coating)	200–400	15.4			“Maximal failure load (N)” calculated from pull-out testing mean + SD measured ( [13]; Fig. 3)	4	Huang (at 4 weeks)
											12	Huang (at 12 weeks)
											24	Huang (at 24 weeks)
Tikhilov et al. 2020 [14]	rabbit	tibia (= trabecular)	rectangular plate l = 10 mm, w = 5 mm, h = 2 mm	“control”	“experimental”	100		450		“Maximum load” (N) calculated from pull-out testing median + IQR reported ( [14]; page 4)	13	Tikhilov (at 13 weeks)

**Table 3:** Characteristics of studies in MA2

Author Publication year	Animal	Implantation site (type of bone)	Implant shape and dimensions (diameter and height)	Controle group (Co) = 3DPPT	Experimental group (Exp) = 3DPPT + post-printing treatment	Type of post-printing treatment	Reported outcome of mechnical testing	follow-up (weeks)	Reference Name in MA 2
Amin Yavari et al. 2014 [15]	rat	femur diafyse (= trabecular)	cross-cut femur defect d outer = 5 mm, d inner = 1,3 mm, h = 6 mm	“AlAcH” (= por Ti (alkali-acid-heat treated))	“AnH” (= por Ti; anodized (also acid- and heat-treated))	oxidation (electro-chemical treatment)	“Maximum torque (N.mm)” mean + SD measured ( [15]; Fig. 8c)	12	Amin Yavari (at 12 weeks)
Garcia-Gareta et al. 2014 [16]	sheep	femur condyle (= trabecular)	cylinder d = 9 mm, h = 11 mm	“No Cells” (= por Ti + CaP coating (3-15 μm))	“With Cells” (= por Ti + CaP coating (3-15 μm) + MSC (Mesenchymal Stem Cells))	adding stem cells	“Maximum Force (N)” from push-out testsing mean + SD kindly provided by autor	6	Garcia-Gareta (at 6 weeks)
Stübinger et al. 2014 [17]	sheep	pelvis (= trabecular)	screw d = 4,1 mm, h = 9 mm	“AM” (= “Additive Manufactured”) (=por Ti (CaF grit blasted + acid washed + US cleaned + dryed))	“AM-PSD” (= AM + dendron functionalisation”) (= por Ti (CaF grit blasted + acid washed + US cleaned + dryed + psd (Phosphoserine modified dendron coating)	adding growth- factors/ drugs/ others	“Removal torque values in Nmm” mean + SD reported ( [17], Table 3)	2	Stübinger (at 2 weeks)
								8	Stübinger (at 8 weeks)
Cohen et al. 2016 [18]	rat	calvaria onlay (= cortical)	cylinder d = 5 mm, h =?	“Rough” (=por Ti (CaF grit blasted + acid etched))	“DBX” (=por Ti (CaF grit blasted + acid etched) + (Demineralized Bone Matrix) puty)	adding bone-matrix	“Force at Failure (N)” from pull-out testing mean + SD measured ( [18], Fig. 3h)	10	Cohen (at 10 weeks)
Liu et al. 2016 [19]	rabbit	tibia (= trabecular)	cylinder d = 5 mm, h = 6 mm	“Simvastatin 0 mg” (=por Ti (US cleaned))	“Simvastatin 0,1 mg” (= por Ti (US cleaned) + 0,1 mg/ml simvastatine/poloxamer 407 hydrogel)	adding growth- factors/ drugs/ others	“Maximum push-in force (N)” mean + SD reported ( [19], Table 4)	4	Liu 1 (at 4 weeks)
								8	Liu 1 (at 8 weeks)
					“Simvastatin 5 mg” (= por Ti (US cleaned) + 0,5 mg/ml simvastatine/poloxamer 407 hydrogel)			4	Liu 2 (at 4 weeks)
								8	Liu 2 (at 8 weeks)
Xiu et al. 2016 [20]	rabbit	femur condyl (= trabecular)	cylinder d = 5 mm, h = 6 mm	“Control” (= por Ti (US cleaned + dried))	“MAO” (= por Ti (US cleaned + dryed) + MicroArc Oxidation)	oxidation (electro-chemical treatment)	“Push out force (N)” mean + SD reported ( [20] section 4 page 10)	8	Xiu (at 8 weeks)
Xu et al. 2016 [21]	rabbit	calvaria (= cortical)	cup with porous scaffold on inside d = 5 mm, h = 5 mm	“native SLM” (= por Ti; US cleaned)	“SLA” (=por Ti; US cleaned + ZrO2 sandblasted + acid etched)	mechano-chemical treatment	“Maximum force (N) to failure of pull-out test” mean + SD measured ( [21], Fig. 9c)	4	Xu 1 (at 4 weeks)
								8	Xu 1 (at 8 weeks)
					“SAN” (= por Ti; US cleaned + ZrO2 sandblasted + anodized)	mechano-chemical treatment		4	Xu 2 (at 4 weeks)
								8	Xu 2 (at 8 weeks)
					“SAH” (=por Ti; US cleaned + ZrO2 sandblasted + alkali heat treated)	oxidation (electro-chemical treatment)		4	Xu 3 (at 4 weeks)
								8	Xu 3 (at 8 weeks)
Bandyopadhyay et al., 2017 [7]	rat	distal femur (= trabecular)	cylinder d = 3 mm, h = 5 mm	“LENS™ Porous Ti” (= “Laser Engineered Net Shaping”)	“LENS™ Porous Ti-NT” (= por Ti, anodised, forming nanotubes (d = 105 nm, l = 375 nm))	oxidation (electro-chemical treatment)	“Shear modulus (MPa)” from push-out testing mean + SD reported ( [7]; Table 3)	4	Bandyopadhyay (at 4 weeks)
								10	Bandyopadhyay (at 10 weeks)
Cheng et al. 2017 [8]	rat	calvaria onlay (= cortical)	block l = 3,5 mm, w = 5 mm h = 2 mm	“Porous” (= por Ti (CaF grit blasted+ acid etched))	“Porous + DBX” (= por Ti (CaF grit blasted+ acid etched) + Decalcified Bone Matrix))	adding bone-matrix	“Pull-out force (N)” mean + SD measured ( [8]; Fig. 4)	10	Cheng (at 10 weeks)
MacBarb et al. 2017 [9]	sheep	distal femur (= trabecular)	triangular prism d = 7 mm (inscribed core circle) h = 45 mm	“AM” (= “Additive Manufactured” por Ti)	“AM + HA” (= por Ti + nanocrystalline HA) 20 nm layer)	adding Ca-containing layer	“Ultimate Shear Strength (MPa)” mean + SD measured ( [9]; Fig. 4a)	6	MacBarb 1 (at 6 weeks)
								12	MacBarb 1 (at 12 weeks)
					“AM + Auto” (= por Ti + autograft)	adding bone-matrix		6	MacBarb 2 (at 6 weeks)
								12	MacBarb 2 (at 12 weeks)
Tanzer et al. 2019 [22]	dog	femur diafyse (= cortical)	cylinder d = 5,2 mm, h = 10 mm	“LRM” (= “Laser Rapid Manufacturing” por Ti)	“LRM-PA” (= por Ti + precipitated HA (20-70 μm layer))	adding Ca-containing layer	“Mean shear strength (Mpa) from push-out testingmean + 95% CI reported ( [22]; results- section, page 3)	4	Tanzer 1 (at 4 weeks)
								12	Tanzer 1 (at 12 weeks)
					“LRM-PSHA” (= por Ti + plasma sprayed HA (20 μm layer))			4	Tanzer 2 (at 4 weeks)
								12	Tanzer 2 (at 12 weeks)
Teng et al. 2019 [23]	rabbit	calvaria (= cortical)	cylinder d = 20 mm, h = 2 mm	“MAO” (= por Ti (sandblasted, acid etched, cleaned, dried) + MicroArc Oxidation)	“MAO-CaP” (= por Ti (sandblasted, acid etched, cleaned, dried) + MAO + CaP	adding Ca-containing layer	“Torsion force (N/cm)” mean + SD reported ( [23], Fig. 6)	6	Teng 1 (at 6 weeks)
								12	Teng 1 (at 12 weeks)
					“MAO-BMP” (= por Ti (sandblasted, acid etched, cleaned, dried) + MAO + CaP + BMP2	adding growth- factors/ drugs/ others		6	Teng 2 (at 6 weeks)
								12	Teng 2 (at 12 weeks)
Bai et al. 2020 [24]	rabbit (OVX)	distal femur (= trabecular)	cylinder d = 6 mm, h = 10 mm	“S” (= “Scaffold” = por Ti (US cleaned))	“SG” (= por ti (US cleaned) + hydrogel)	adding growth- factors/ drugs/ others	“Push-out force (N)” mean + SD measured ( [24] Fig. 6c)	12	Bai 1 (at 12 weeks)
					“SGC” (= por ti (US cleaned) + hydrogel + stem cells)	adding stem cells		12	Bai 2 (at 12 weeks)
					“SGB” (= por ti (US cleaned) + hydrogel + BMP2	adding growth- factors/ drugs/ others		12	Bai 3 (at 12 weeks)
					“SGCB” (= por ti (US cleaned) + hydrogel + stem cells + BMP2)	adding stem cells		12	Bai 4 (at 12 weeks)
Fan et al. 2020 [25]	rabbit	radius (= trabecular)	cylinder d = 5 mm, h = 13 mm	“pTi” (= por Ti)	“BaTiO3/pTi (= porTi + BaTiO3 piezoelectric coating)	adding growth- factors/ drugs/ others	“Peak pull out load (N)” mean + SD measured ( [25] Fig. 11c)	6	Fan 1 (at 6 weeks)
								12	Fan 1 (at 12 weeks)
					“pTi + LIPUS” (= por Ti + Low-Intensity Pulsed Ultrasound treatment)			6	Fan 2 (at 6 weeks)
								12	Fan 2 (at 12 weeks)
					“BaTiO3/pTi” (= porTi + BaTiO3 coating + Low-Intensity Pulsed Ultrasound treatment)			6	Fan 3 (at 6 weeks)
								12	Fan 3 (at 12 weeks)

#### Quality of research

**Fig. 2:**
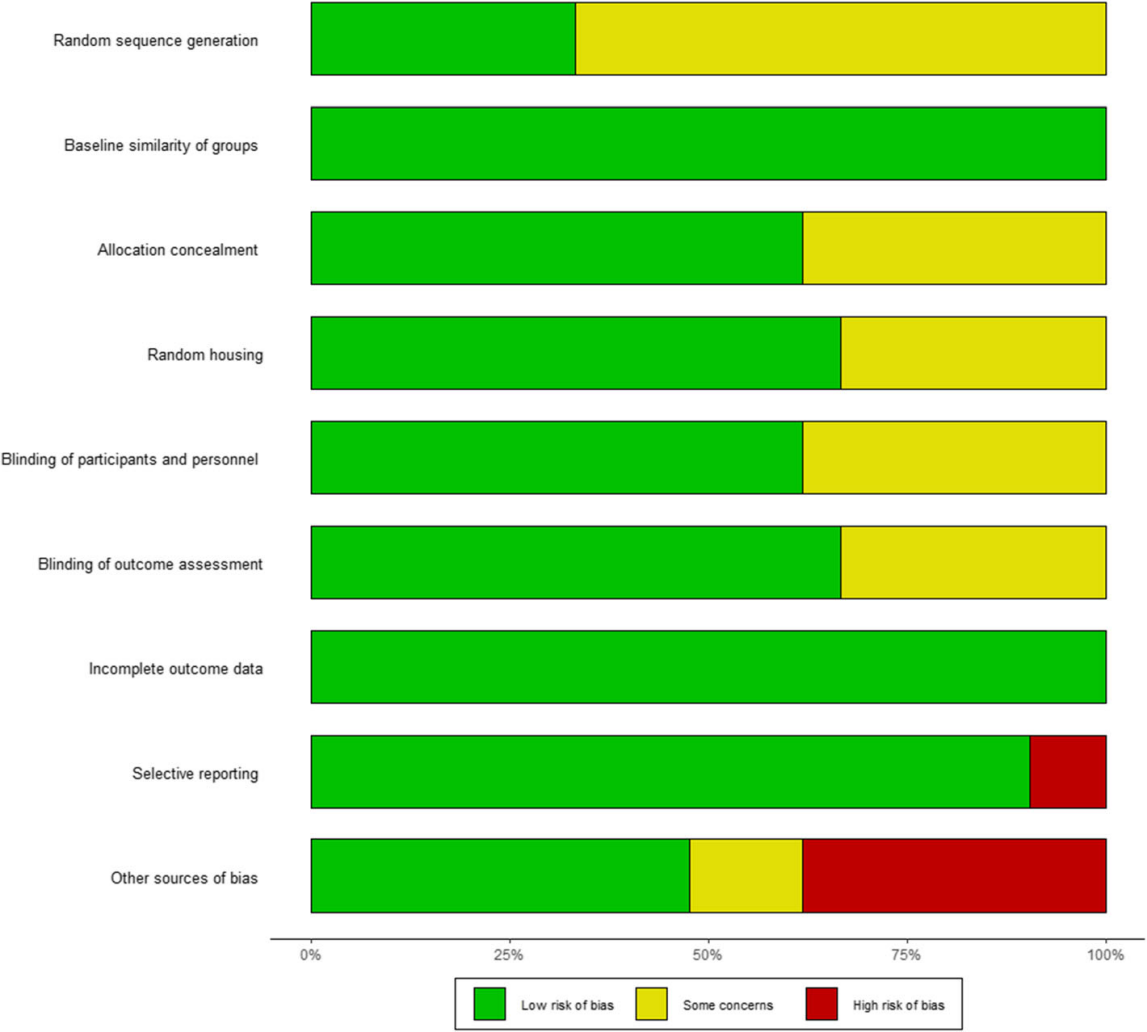
ROB Plot

The results of the ROB evaluation are shown in Fig. 2. Our studies showed only few actual predispositions toward bias (namely one case of selective reporting [12]). Furthermore, eight studies mentioned financial support of industry partners. In general, “random sequence generation”, “blinding of personnel”, and “allocation concealment” were poorly reported, causing uncertainty. Most publications mentioned institutional approval of study protocols and concordance to institutional guidelines concerning animal use and care.

#### Results of quantitative synthesis

**Fig. 3:**
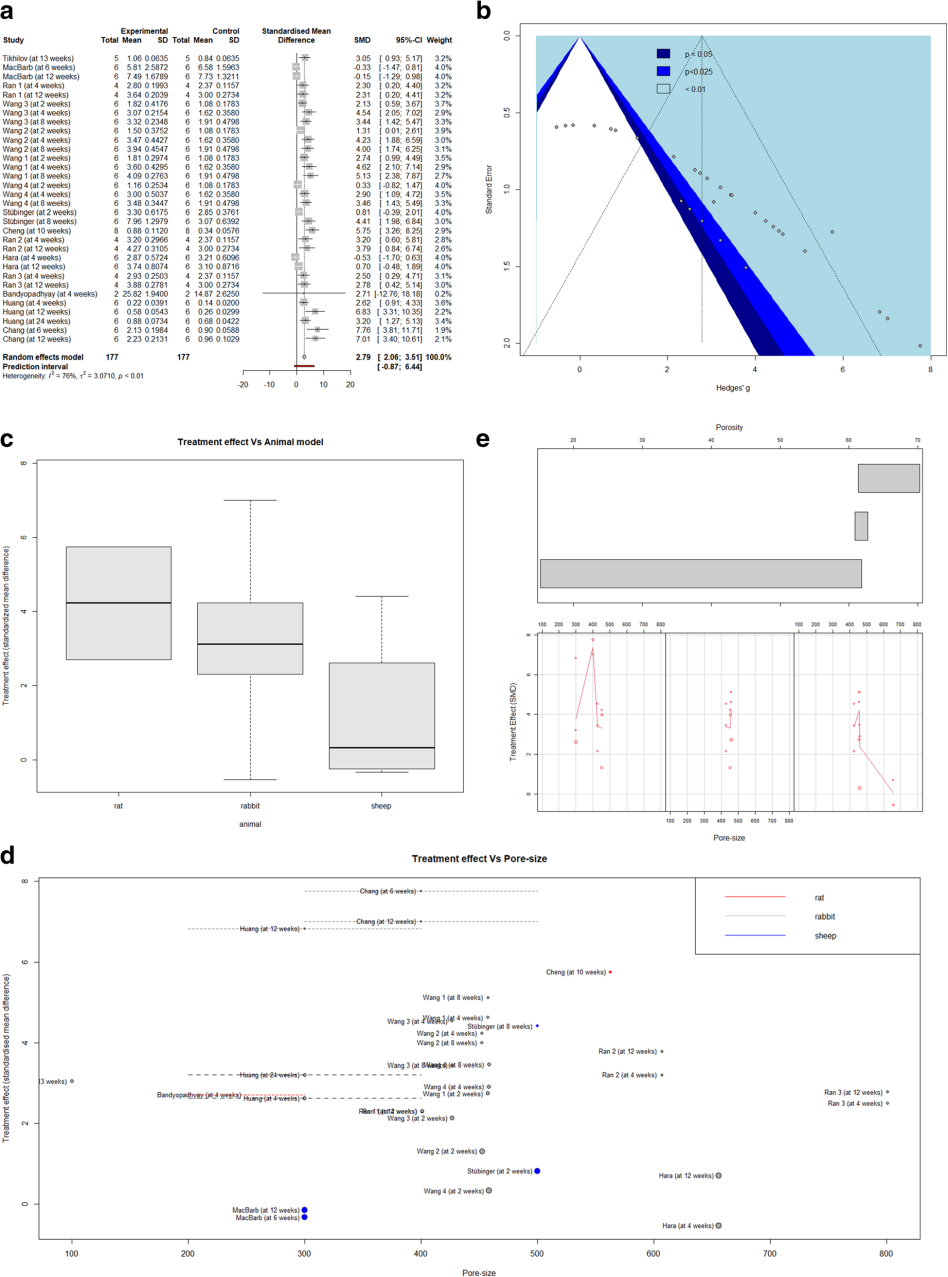
Graphics from MA1: **a** Forest plot displaying included studies and calculated treatment effects (SMD). **b** Funnel plot displaying publication bias. **c** Regression model of moderator analysis 1 showing the relationship between the treatment effect and the moderator “animal type”. **d** Pore-sizes of all the studies included in MA1 (weighted with the inverse of their variance for their size, represented with a dotted line in the case of the reported pore-size range) vs. their corresponding calculated TE. **e** Coplot displaying pore-sizes of all the studies included in the rabbit subgroup analysis vs. their corresponding TE (SMD) (below), but allocated to three graphs, corresponding to three clusters of porosity values (above)

The studies included in MA1 were all very small and showed considerable heterogeneity (I^2^ = 76.3%; i.e., the percentage of variability in ES not caused by sampling error). Thus, we applied a mixed-effects model, which offered a between-study variance estimate (τ^2^) of 3.0710 (CI [1.3639; 5.8734]) and estimated the SMD (of the shear strength of the bone-titanium interface between the 3DPPT and dense Ti groups) to be 2.79 (*p* < 0.0001 and CI [2.0613; 3.5090]). The forest plot of MA1 is displayed in. 3a and shows that most of the studies have a positive TE with widespread overlap in CIs. Removing statistical outliers from the MA (a total of 6 observations, for which the CIs did not lie within the CI of the pooled effect) elevated the SMD to 3.39 (and lowered the I^2^ to 24). However, because there was no evidence that these study results were invalid for our study, we did not exclude these outliers. Figure 3b shows the funnel plot of MA1, displaying noticeable asymmetry (with confirmation by Egger’s test).

To extend this MA to a meta-regression, we first conducted an exploratory AICc-based ranking of moderators but ultimately performed step-down selection based on the offering of a significant *p*-value for the test of moderators. Our final (most appropriate) model only included the predictor “animal type” as the independent variable [test of moderators F (df1 = 2, df2 = 293.8722), *p* = 0.0323, *R*^2^ = 33.24%]. In this model, the regression coefficient (RC) for the animal type “rabbit” was estimated to be 2.7 times higher than for the “rat” model (intercept = 5.6009) and the “sheep” model was estimated to be 4.7 times lower. These findings are shown in Fig. 3c. Extending this model to multivariable meta-regression by adding “pore-size” as a covariable also yielded a significant *p*-value for the test of moderators [F (df1 = 3, df2 = 28) = 3.0419, *p* = 0.0453] as well as that of a model involving the interaction of “pore-size” and “animal type”[F (df1 = 5, df2 = 26) = 2.8876, *p* = 0.0333]. These combinations raised the accounted heterogeneities (R^2^) to 38.55 and 52.78%, respectively. However, the likelihood ratio test comparing all three models found no superiority of the extended models; thus, the simpler “animal type only” model was favored. To offer a better understanding of their relationship, Fig. 3d shows the TE of all observations in MA1 as a function of the variable “pore-size” reported in the study.

Because “rabbit” was our most represented model, we performed subgroup analysis to exclude the aforementioned effect of “animal type” and further investigate the role of other possible moderators. Here, a multivariable regression model with the interaction of covariables “pore-size” and “porosity” provided the most significant *p*-value (*p* = 0.0130) for the test of moderators [F (df1 = 3, df2 = 15) = 5.0371], with the likelihood ratio test favoring it over a reduced “pore-size only” or “pore-size and porosity but no-interaction” model. We noted that “pore-size” and “porosity” were correlated (0.79), making it impossible to fully distinguish between both. This model accounted for 66.44% of the heterogeneity (R2) and RC = − 0.0022 [*p* = 0.0803 (not significant, but strongly associated)]. This finding is illustrated in Fig. 3e, which shows a coplot of the covariates “pore-size” and “porosity”.

**Fig. 4:**
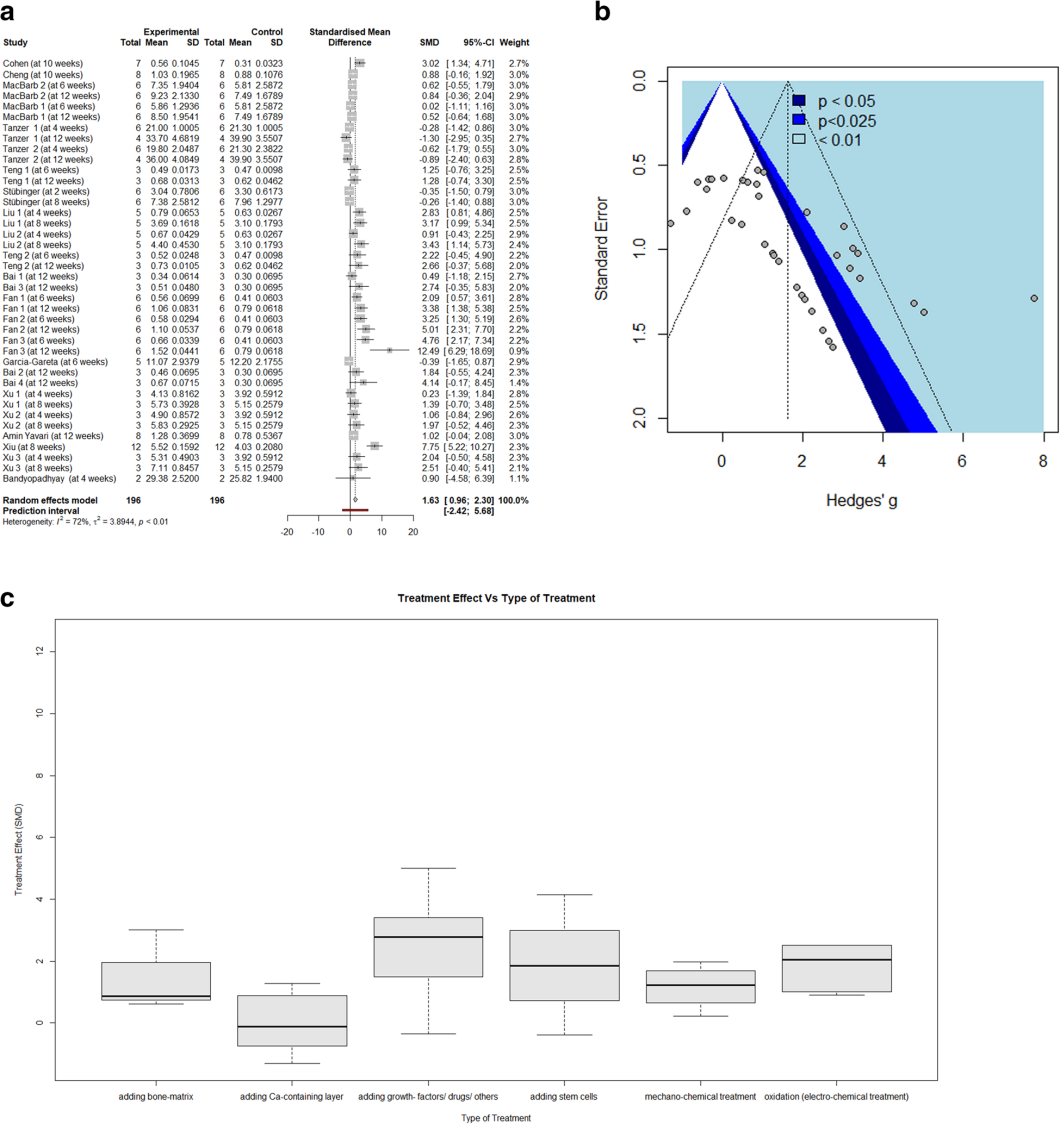
Graphics from MA2: **a** Forest plot displaying the included studies and calculated treatment effects (SMD). **b** Funnel plot displaying publication bias. **c** Regression model of moderator analysis 2 showing the relationship between the treatment effect and the moderator “type of treatment”

MA2 showed a similar, moderately high heterogeneity (I^2^ = 72.2%) and a between-study variance (τ^2^) of 3.8944 (CI[1.5477; 6.5609]) for which a mixed-effects model was applied again. The data, in turn, provided an SMD of 1.63 (*p* < 0.0001, CI [0.9612; 2.2975], PI [− 2.4212; 5.6799]). The forest plot of MA2 is shown in Fig. 4a and shows even greater overlap in CIs than that of MA1. Removing outliers [10] would have lowered I^2^ to 36.6% and kept the SMD at 1.64 (while narrowing the CI to [1.2036; 2.0893]. However, we excluded this procedure because we had no evidence that these study results did not affect our studies. Similar to MA1, MA2 showed noticeable funnel plot asymmetry, displayed in Fig. 4b.

Regarding MA2, we included the single moderator “treatment type” to provide the model with the best *p*-value for the test of moderators (F (df1 = 5, df2 = 34) = 2.5932, *p* = 0.0432). The likelihood ratio test favored this model over a combined “animal type and treatment type” model, and it provided an R^2^ of 44.58%. The RCs for the individual “treatment types” were not significant.

## Discussion

Many reports have been published concerning 3DPPT [6, 9, 12, 14, 25], and reviews have revealed the difficulty of decision making based on the interpretation of this information [26–28]. Publications attempting to provide an overview almost always rely on either a biomimetic approach (3DPPT printed so that the lattice provides an optimized mechanical and biological match to bone tissue) or optimal printability/producibility. When 3DPPT is approached from this biomimetic starting point, ellipsoidal pores of 300–600 μm should match cancellous bone and cylindrical canals of pore-size 10–50 μm should match cortical bone [29]. The porosities of cancellous and cortical bone are 50–90% and 3–10%, respectively. However, in 3DPPT, “porosity” is more often approached as the parameter to adjust the Young’s modulus and is varied in the function of desired mechanical stiffness. A recent review by Martinez-Marquez et al. on experimental 3DPPT research reported the three most used types of unit cells as “diamond”, “gyroid TPMS”, and “cubic”, with 56.6% of studies using a porosity of 30–70% and 86.8% applying pore sizes between 100 and 1000 μm [29]. Some studies experimenting within these ranges have stated that, for cell ingrowth, a 100-μm pore-size diameter would be a minimum value; for vascular invasion and the formation of capillaries, this value would more likely be approximately 300 μm [12]. The ideal ranges for OI are most often defined between 200 and 400 μm [9] and 50–400 μm for soft-tissue integration [14]. Larger diameters would permit better initial cell migration and nutrient diffusion but rapidly diminish mechanical strength [25]. Studies then comparing different pore sizes at a set porosity have found superior pull-out strength of pore-size 600 μm over 300 μm and 900 μm in rabbits at a 2-week observation point [30]. Wang et al. used implants with similar pore sizes, Struth sizes and porosities but varying distributions and configurations of unit cells and found no significant differences in the pullout strength in rabbits at either time point [11]. Li et al. also confirmed these findings, applying gradients of pore sizes 300–500 μm, 200–600 μm, and 100–700 μm for a 5-week observation in mini-pigs [25].

Here, we conducted an SR and 2 separate MAs. The SMDs calculated (2.79 for MA1 and 1.63 for MA2) were not the focus of our search. They express a difference in the mechanical removal strength between porous and non-porous 3D-printed titanium implants and between post-treated and non-post-treated 3DPPT implants, expressed as the difference in the of SDs of TE; these differences are reasonable and have never been challenged, particularly concerning the former. However, the asymmetry in the funnel plots (Figs. 3b and 4b) should temper these assumptions. Because the funnel plots display a negative, almost linear regression between the standard error (SE) and treatment effect (TE) (i.e., large treatment effects are reported by less precise (smaller) studies, while more reliable (large) studies show little to no TE), it appears that the results of our MAs show evidence of publication bias.

In MA1, we found that the “animal model” as a moderator may explain a difference in TE. Because our TE indicates improvement in the mechanical removal strength when making the implants porous vs not porous, less improvement is expected when implanting a porous implant in a sheep than in a rat. For the rabbit model, we had many more observations than those for rats or sheep (26/32 observations), and the TE values enclosed a wider reach (Fig. 3d; observations in gray). Here, the best fitting model involved the interaction of “porosity” and “pore-size”. The strong relationship between these covariables (correlation coefficient of approximately 0.8) reflects that these factors may not be fully distinguishable characteristics of the included studies [31]. Regarding content and in a context of 3DPPT, the influence of “pore-size” on mechanical removal strength should be investigated considering the number of pores. Thus, we included “porosity” to represent this measure, although it is more accurately a measure of the porous volume fraction; our MAs do not allow us to derive an accurate estimate of the number of pores for each lattice reported. For the actual representation of these covariables, we used a coplot, displaying the regression-lines of “pore-size” to “TE” at three corresponding (but overlapping) ranges of “porosity” (Fig. 3e). In summary, the three curves formed describe the following: 1) an ascent, with a peak at a “pore-size 400 μm” and then a decline in the TE of implants with a “pore-size” of 300–500 μm and a “porosity” of 15–61.6%; 2) a slight decline and again sharp ascent in the TE of implants with a “pore-size” of 400–500 μm and a “porosity” of 61.1–66.2%; and 3) a small peak, rapid decline at a “pore-size” of 400–500 μm and a slower decline (reaching TE = 0) at a “pore-size” > 500 μm and a “porosity” of 62.5–70%. Our study found the highest TE at a pore size of 400 μm and a porosity of 55%; however, considering precision, it is likely safer to conclude that a pore-size of 300–450 μm, with a porosity of 50–65%, consistently shows improved OI (Fig. 3e), at least in a rabbit model. We have no observations at less than 300 μm but observed a decline at greater than 450 μm. Literature describing tested animal models states that the bone microanatomy of rabbits and sheep differ from that of the humans and also from each other; for example, the average diameter of the long bone trabecula of sheep is less than 100 μm and that of rabbits is 50–220 μm [32]. Walsh et al. [33] observed significantly more bone ingrowth in 3DPPT implanted in a cortical sheep tibia model than in a trabecular femur model, indicating that the implantation site might be important for OI. Garcia-Gareta et al. [16] also noticed a reduced push-out strength of 3DPPT implanted in a sheep gap model vs. a press-fitted model (regardless of the addition of stem cells). However, to our knowledge, no study has explored differences in the mechanical strength of the OI of 3DPPT using scaffold parameters more closely adjusted to the animal (bone) type or implantation site.

In MA2, the only significant moderator was “type of treatment”, which showed that the treatments comprising adding a Ca-containing layer had a significantly lower TE than the other treatment types. This finding is likely controversial because the beneficial effect of these types of coatings (CaP, hydroxy-apatite) on the bone-titanium interface strength of solid titanium is well established [34–37]. A possible explanation could be sought for the supposed pore-filling effect of the Ca-containing layer that might negatively affect the mechanical bone-titanium interface in short-term evaluations. Because these Ca-containing layers are biodegradable and diminish over time, a supposed changed influence over time (exceeding the 6-month maximum span of our reported studies) might also be worth exploring.

However, because certain caveats apply to our study, our findings should be interpreted with caution. First, substantial heterogeneity was found among the included studies, as demonstrated by the high I^2^ values. An explanation could be the discrepancy between grouping different animal models at the MA level and extreme genetic similarity of the individual animals at the study level [4]. The Cochran Q value in the rabbit subgroup analysis was lower (85; *p* < 0.0001) but still significant. The SMDs from MA1 and MA2 both show high between-study variance values, indicating that the estimated effects differ across studies. Another explanation could be an overlooked effect due to variation in the observation methods (i.e., non-correspondence of pull-out, push-out, and torque-out testing). However, because the included studies were small (12 samples at best), there is no straightforward explanation for the heterogeneity and we should conclude that both MAs are sensitive to biasing animalities [38]. Second, our allocation of studies between the two MA pools is debatable. In MA1, in the study of Huang et al., the Exp group was porous but also HA coated (1-μm thick) [13]. However, our study considered 1 μm as a thin layer [36]. In MA2, the allocation of “similar types of post-treatment” into six categories could not be performed completely unambiguously because some studies had characteristics compatible with multiple categories [24]. Third, because MA1 and 2 comprised sample sizes of 177 and 196 animals in 32 and 40 observations, respectively, we had sufficient samples to perform MAs. With this level of observation, applying models with up to two predictors and an interaction effect is considered a valid practice [4]. However, multivariable meta-regression of the rabbit subgroup analysis was based on only 19 observations, which might be at the lower limit of what is acceptable [4]. A full comparison of all forementioned moderators was restricted to only 12 observations (studies always reported “pore-size”, and most often only “porosity” or “Struth-size”, seldomly both, and many authors did not respond to our requests for extra specification). The final caveat, though obvious, would be not to confuse statistical associations for causality.

## Conclusion

We performed two separate MAs with moderator analyses to determine whether statistical models including reported scaffold features (“pore-size”, “porosity”, and” type of unit cell”) or post-printing treatments (adding stem cells, growth factors, drugs, and surface treatments) could explain the observed differences in the treatment effect. Our findings suggest a stronger effect from porosity in a rat model than that in a sheep model. Additionally, adding a calcium-containing layer does not improve the mechanical removal strength but the other post-treatment types do. Our results provide an overview and some new insights but little narrowing of the existing value ranges. We would advocate more research involving comparing implantation to similar, “standardized” control groups and expressing “pore-size” and “porosity” in terms of surface roughness to help address existing dilemmas, along with the consequent reporting of 3DPPT characteristics.

## Data Availability

The datasets used and/or analyzed during the current study are available from the corresponding author on reasonable request.

## Abbreviations

Ti: Titanium
Ta: Tantalum
Ca: Calcium
n: Number
3DPPT: 3D-Printed, Porous Titanium
OI: Osteointegration
SR: Systematic Review
MA: Meta-Analysis
ROM: Range of Motion
Exp: Experimental Group
Co: Control Group
SD: Standard Deviation
SMD: Standardized Mean Differences
CI: 95% Confidence Interval
τ^2^: Between-study Variance
PI: Prediction Interval
OVX: Ovariectomy
TE: Treatment Effect
ROB: Risk of Bias

## Footnotes

1 Exp and Co had the same composition as Ti, with identical implant shape and dimensions, implanted in the same way, at the same location, in the same animal model.

2 Same as ^1^, but Exp and Co also shared the same design parameters (“pore-size”, “porosity”, “unit cell”, and “Struth-size”) for their porous structure.
